# Endoscopic ultrasound-guided gallbladder drainage and electrohydraulic lithotripsy for the treatment of gallbladder neck stones

**DOI:** 10.1055/a-2808-6901

**Published:** 2026-03-02

**Authors:** Yifan Wang, Qianqian Dong, Lingmin Li, Xiaofeng Liu, Wenbo Li, Qun Li

**Affiliations:** 1 Department of Gastroenterology, The 960th Hospital of the PLA Joint Logistics Support Force, Jinan, China; 2 Shandong First Medical University, Jinan, China

A 32-year-old man presented with recurrent right upper abdominal pain for >1 year, exacerbated by fatty food intake. Ultrasonography indicated gallbladder stones (22 mm × 8 mm) complicated with cholecystitis. He was admitted for the treatment of gallbladder stones. After admission, due to impacted stones in the gallbladder neck, the gallbladder contraction test yielded no measurable results, while laboratory examination indicators showed no obvious abnormalities. Given the patient's strong desire for gallbladder preservation, endoscopic ultrasound-guided gallbladder drainage (EUS-GBD) was performed on 19 September 2025. Endoscopic lithotripsy was conducted 1 month later ( Video 1 ). The endoscope was advanced along the stent, revealing impacted stones in the gallbladder neck ( Fig. 1 ). Repeated attempts using a snare, lithotripsy basket, and stone extraction basket were unsuccessful in removing the stones. Therefore, endoscopic electrohydraulic lithotripsy (EHL) under direct vision was adopted to fragment the stones, followed by successful extraction. On postoperative day 4, endoscopic re-examination confirmed a clear gallbladder lumen without residual stones, and the AXIOS stent was removed ( Fig. 2 ). Gastroscopic re-examination 1 week after the operation indicated that the fistula orifice had basically healed. Two months after surgery, the gallbladder contraction test showed good function ( Fig. 3 ).

Endoscopic ultrasound-guided gallbladder drainage and electrohydraulic lithotripsy for the treatment of gallbladder neck stones.Video 1

**Fig. 1:**
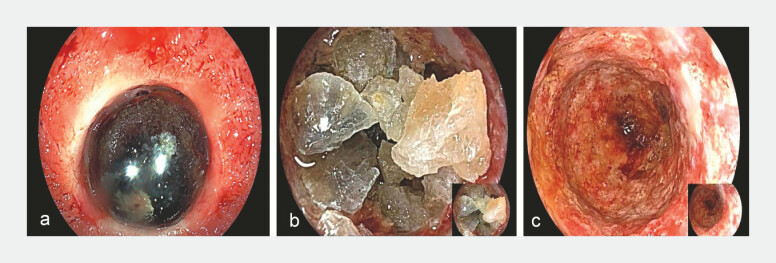
The combination of endoscopic ultrasound-guided gallbladder drainage and electrohydraulic lithotripsy. **a** Impacted gallbladder neck stones. **b** Electrohydraulic lithotripsy. **c** Clean gallbladder lumen after stone extraction.

**Fig. 2:**
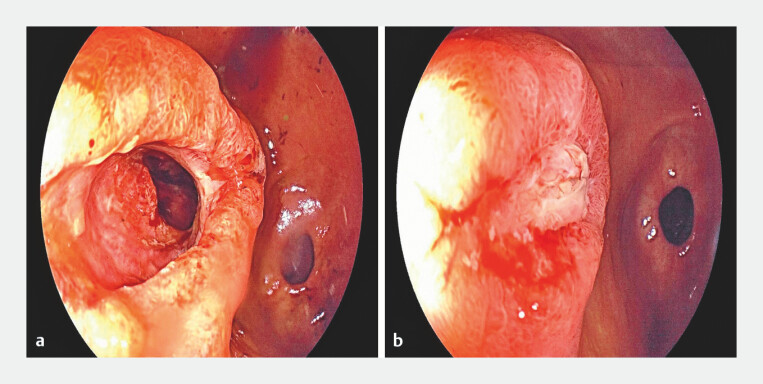
Post AXIOS stent extraction and fistula healing status. **a** Post stent removal. **b** Nearly closed fistula with local ulceration 1 week postoperatively.

**Fig. 3:**
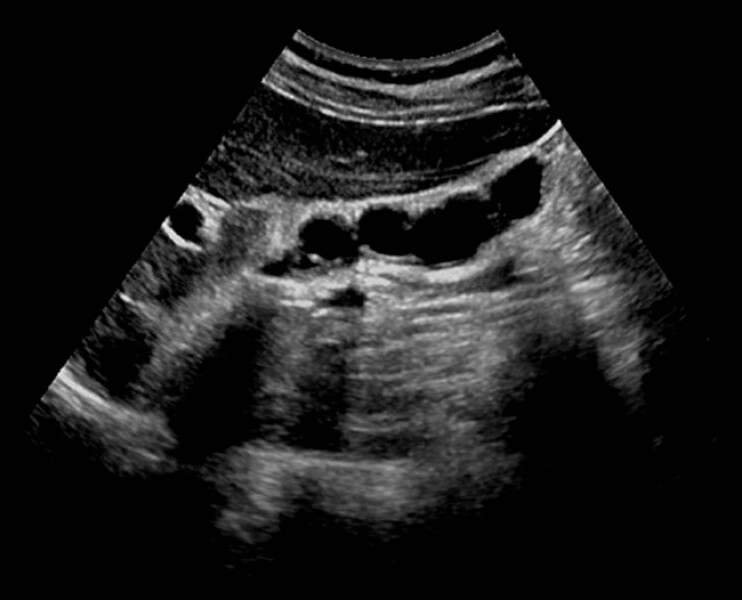
The gallbladder contraction test performed 2 months after surgery showed good gallbladder contractile functions.

Gallbladder stones are common with rising incidence, 1 which can lead to complications such as acute cholecystitis, acute pancreatitis, gallstone ileus, and gallbladder cancer. 2 Current treatment modalities usually include EUS-guided interventional stone extraction and surgical intervention. 3 At present, EHL is widely used in the treatment of biliary tract stones under surgical choledochoscopy, characterized by high lithotripsy efficiency and minimal tissue damage 4 . Previously, impacted gallbladder neck stones generally required surgical cholecystectomy. This case report describes the first innovative application of EUS-GBD combined with electrohydraulic lithotripsy for the treatment of impacted gallbladder neck stones, ultimately achieving complete stone clearance while preserving gallbladder functions.

Endoscopy_UCTN_Code_TTT_1AS_2AH

## References

[1] Song Y Ma Y Xie F Age, gender, geographic and clinical differences for gallstones in China: a nationwide study Ann Transl Med 2022 10 735 10.21037/atm-21-6186 35957733 PMC9358507

[2] Baca-Arzaga A Navarro-Chávez A Galindo-Jiménez A Gallstone lithotripsy with SpyGlass system through a cholecystoduodenal fistula in a patient with type IIIa Mirizzi syndrome Rev Gastroenterol Mex (Engl Ed) 2021 86 99 101 10.1016/j.rgmx.2020.01.003 32522464

[3] Chan S Teoh A Yip H Feasibility of per-oral cholecystoscopy and advanced gallbladder interventions after EUS-guided gallbladder stenting (with video) Gastrointest Endosc 2017 85 1225 1232 10.1016/j.gie.2016.10.014 27756612

[4] Manes G Paspatis G Aabakken L Endoscopic management of common bile duct stones: European Society of Gastrointestinal Endoscopy (ESGE) guideline Endoscopy 2019 51 472 491 10.1055/a-0862-0346 30943551

